# Prevalence and risk factors for long COVID after mild disease: A cohort study with a symptomatic control group

**DOI:** 10.7189/jogh.13.06015

**Published:** 2023-05-12

**Authors:** Ana B Cazé, Thiago Cerqueira-Silva, Adriele P Bomfim, Gisley L de Souza, Amanda CA Azevedo, Michelle QA Brasil, Nara R Santos, Ricardo Khouri, Jennifer Dan, Antonio C Bandeira, Luciano PG Cavalcanti, Manoel Barral-Netto, Aldina Barral, Cynara G Barbosa, Viviane S Boaventura

**Affiliations:** 1 Federal University of Bahia, Salvador, Bahia, Brazil; 2 Gonçalo Moniz Institute, Fiocruz, Salvador, Bahia, Brazil; 3 Bahia School of Medicine and Public Health, Salvador, Bahia, Brazil; 4 Santa Izabel Hospital, Santa Casa da Bahia, Salvador, Bahia, Brazil; 5 Department of Health Surveillance/Epidemiological Surveillance, Campo Formoso, Bahia Brazil; 6 Department of Microbiology, Immunology and Transplantation, Rega Institute for Medical Research, Laboratory of Clinical and Epidemiological Virology, Belgium; 7 Center for Infectious Disease and Vaccine Research, la Jolla Institute for Immunology (LJI), San Diego (UCSD), California, USA; 8 Department of Medicine, Division of Infectious Disease and Global Public Health, University of California, San Diego (UCSD), California, USA; 9 Faculty of Technology and Sciences of Salvador, Salvador, Bahia, Brazil; 10 Central State Laboratory Lacen/BA, Salvador, Bahia, Brazil; 11 Federal University of Ceará, Ceará, Brazil; 12 Christus University Center, Fortaleza, Ceará, Brazil; 13 Institute for Research in Immunology, São Paulo, São Paulo, Brazil

## Abstract

**Background:**

There is limited data on the prevalence and risk factors for long COVID and few prospective studies with appropriate control groups and adequate sample sizes. We performed a prospective study to determine the prevalence and risk factors for long COVID.

**Methods:**

We recruited individuals aged ≥15 years who were clinically suspected of having an acute SARS-CoV-2 infection from September 2020 to April 2021. We collected nasopharyngeal swabs three to five days following symptom onset for analysing using reverse transcriptase polymerase chain reaction (RT-PCR). We also collected clinical and sociodemographic characteristics from both SARS-CoV-2 positive and negative participants using structured questionnaires. We followed-up the participants via telephone interview to assess early outcomes and persistent symptoms. For COVID-19 cases, 5D-3L EuroQol questionnaire was used to assess the impact of symptoms on quality of life.

**Results:**

We followed 814 participants (412 COVID-19 positive and 402 COVID-19 negative persons). Most (n = 741/814) had mild symptoms. Both groups had similar sociodemographic and clinical characteristics, except for the hospitalization rate (15.8% in the COVID-19 positive vs 1.5% in the COVID-19 negative group). One month after disease onset, 122/412 (29.6%) individuals in the COVID-19 positive (long COVID) and 24 (6%) in the COVID-19 negative group reported residual symptoms. In the long COVID group, fatigue, olfactory disorder, and myalgia were the most frequent symptoms in the acute phase. Compared to recovered individuals, older age and having more than five symptoms during the acute phase were risk factors for long COVID. Quality of life was evaluated in 102 out of 122 cases of long COVID, with 57 (55.9%) reporting an impact in at least one dimension of the European Quality of Life 5 Dimensions 3 Level (EQ-5D-3L) questionnaire.

**Conclusions:**

In this prospective study consisting predominantly of individuals with mild disease, the persistence of symptoms after an acute respiratory illness was associated with a diagnosis of COVID-19. Polysymptomatic acute disease and older age were risk factors for long COVID.

Long COVID has received less attention from the scientific community than acute COVID-19, representing less than 2% of COVID-19 publications in PubMed Clinical Queries (as of August 31, 2022). Consequently, several important aspects remain undefined, such as its prevalence. Long COVID has been reported to affect between 7.5% to 89% of patients [1-4]. However, most of these studies do not include well-matched controls, which is essential because long COVID symptoms are non-specific and may be attributed to other factors such as the physical and mental impact of having COVID-19 infection or hospitalization [5,6]. Prospective studies enrolling symptomatic individuals with and without confirmed COVID-19 are necessary to better estimate the prevalence of long COVID and its associated risk factors.

Identifying risk factors for developing long COVID is key to mitigating its anticipated impact on the healthcare systems [7]. Some large cohorts using previously hospitalized patients reported risk factors for developing long COVID [1,8,9]. However, few studies evaluated risk factors in individuals after mild disease [10]. We examined the clinical characteristics and diagnosis of all patients presenting with acute respiratory illness at three healthcare units in Bahia-Brazil and the prevalence of long COVID in the SARS-CoV-2 positive group. Additionally, we explored the risk factors associated with this condition and its impact on the participants’ quality of life.

## METHODS

### Study population

We recruited individuals aged ≥15 years who were suspected of having a SARS-CoV-2 infection from healthcare units in three municipalities of Bahia State, Brazil (Irecê, Campo Formoso, and Lauro de Freitas). We excluded individuals who had difficulty reporting symptoms, who were at >20 days post-symptom onset at the time of recruitment, or who previously reported a SARS-CoV-2 infection. The recruitment period lasted from September 2020 to April 2021. The ancestral strain or gamma variant of SARS-CoV-2 was dominant in Brazil from November 2020 to August 2021 (Figure S1 in the **Online Supplementary Document**) [11].

### Exposure measurement

We collected nasopharyngeal swab samples from all patients to test for SARS-COV-2 using reverse transcriptase polymerase chain reaction (RT-PCR). We excluded patients who had an RT-PCR ten days following symptom onset. We collected the samples using either the BIOMOL OneStep/COVID-19 or the Allplex SARS-CoV-2 Assay testing kit, with a sensitivity above 90% and specificity above 97% [12,13]. We reported the results following the Strengthening the Reporting of Observational Studies in Epidemiology (STROBE) (**Online Supplementary Document**) [14].

### Recruitment strategy and data collection

Consecutive in-person recruitment included the application of a structured survey (Questionnaire 1 and Figure S2 in the **Online Supplementary Document**) using the Research Electronic Data Capture (REDCap) software hosted by the Gonçalo Moniz Institute (IGM/FIOCRUZ) in Bahia, Brazil. We collected information on sociodemographic characteristics (age, gender, skin colour, body mass index, anthropometric data, municipality of residence), comorbidities (hypertension, diabetes, heart disease, chronic lung disease, other), date of symptom onset, acute symptoms (fever, myalgia, arthralgia, cough, shortness of breath, nasal congestion, sneezing, coryza, sore throat, sinus pain, retroocular pain, ear pain, headache, chest pain, chest discomfort, rash, abdominal pain, loss of appetite, vomiting, diarrhoea (defined as two or more episodes in the last 24 hours), weakness, fatigue, light-headedness or fainting, loss of smell, and loss of taste).

### Follow-up and outcome measurement

We followed-up on SARS-CoV-2 positive (COVID-19) and negative (non-COVID-19) patients who reported COVID-19-like symptoms, recruiting both on the same day prior to nasopharyngeal swab sampling. We considered the date of symptom onset as day zero. We conducted a second structured questionnaire (Questionnaire 2 and Figure S3 in the **Online Supplementary Document**) 30 to 60 days after disease onset via telephone to document the RT-PCR result, acute symptoms, and clinical outcomes. We classified participants as non-hospitalised or hospitalised in the intensive care unit (ICU) or non-ICU. We applied Questionnaire 2 up to 40 days after recruitment (Questionnaire 1) to capture any symptoms in the acute phase.

We conducted a third structured questionnaire 60 to 250 days after disease onset by telephone to evaluate residual symptoms (Questionnaire 3 and Figure S4 in the **Online Supplementary Document**). The questions regarded residual or new symptoms: cough, fatigue, shortness of breath, headache, chest pain, dysphonia, dysphagia, loss of appetite, loss of smell, loss of taste, myalgia, arthralgia, fever, and other non-listed symptoms. We excluded patients reporting suspected or confirmed re-infection after the recruitment. Figure S2 in the **Online Supplementary Document** shows the flow of an individual after recruitment.

### Outcomes of interest

We defined long COVID according to the Centers for Disease Control and Prevention classification [15] and persistence of symptoms as the presence of at least one residual symptom >1 month post-disease onset. The list of evaluated residual symptoms (Questionnaire 3) was based on previous studies [16,17].

We applied the European Quality of Life 5 Dimensions 3 Level (EQ-5D-3L) questionnaire via telephone to assess health dimensions: mobility, self-care, daily activities, pain/discomfort, and anxiety/depression. We also used the European Quality visual analogue scale (EQ-VAS), with 0 rated as the worst and 100 as the best imaginable health state [18]. We applied the Portuguese-validated version of the EQ-5D-3L questionnaire [19].

### Statistical analysis

We described categorical data using counts and percentages and continuous data (determined using the Shapiro-Wilk test) using medians and interquartile ranges (IQRs). We constructed the correlation matrix based on the frequency of reported residual symptoms. We performed multivariable logistic regression to explore risk factors previously associated with long COVID in the literature: age, sex, and number of symptoms in the acute phase [10,20-22]. We also performed a sensitivity analysis, including on the comorbidities. The estimated 95% Wald confidence interval (95% CI) was employed for measures of association to interpret the findings. We used the R statistical software (version 4.2.0) for all analyses [23].

### Ethics

We obtained informed consent from all participants. The Ethics in Research Committee of the Gonçalo Moniz Research Center approved this study (approval No. 4.315.319/202).

## RESULTS

**Figure 1:**
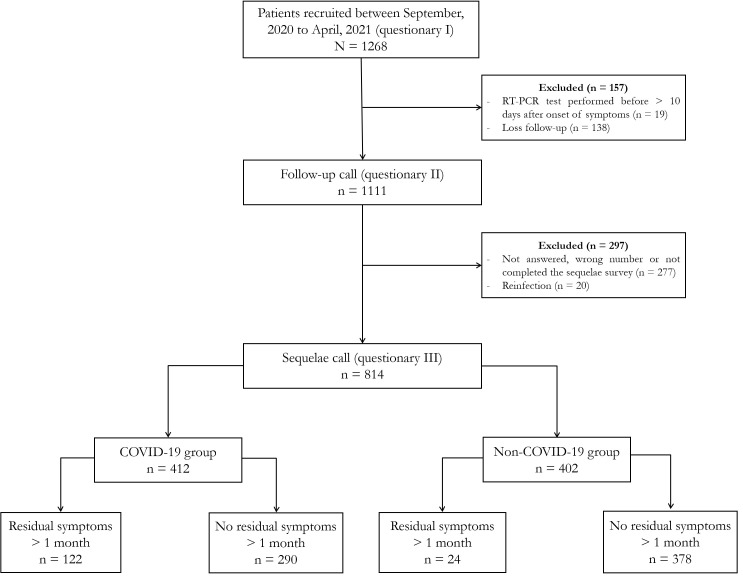
Flowchart of the study population. RT-PCR – reverse transcription polymerase chain reaction.

We recruited 1268 patients with suspected SARS-CoV-2 infection up to five days post-disease onset, all of whom answered the first questionnaire. We excluded 39 (3.0%) individuals who were tested outside RT-PCR recommended period or reported a second SARS-CoV-2 infection and 415 (32.8%) who were lost to follow-up. A total of 814 (64.2%) patients were considered eligible for the analysis of residual symptoms (**Figure 1** and Figure S5 in the **Online Supplementary Document**). Questionnaires 2 and 3 were applied 40 (IQR = 31-59) days and 102 (IQR = 63-150) days post-symptom onset, respectively. We observed no difference in sociodemographic data and frequency of positive RT-PCR between the included (n = 814) and excluded (n = 454) participants **(**Table S1 in the **Online Supplementary Document**). Among the included participants, 412 tested positive (COVID-19 group) and 402 tested negative (non-COVID-19 group) for SARS-CoV-2. The COVID and non-COVID groups had a median age of 36 (IQR = 28-48) and 34 (IQR = 25-43) years, respectively. Furthermore, 55% of the participants in the COVID group and 64% in the non-COVID group were female. Eighty-four per cent of the participants in the COVID group and 99% in the non-COVID group had mild symptoms (**Table 1** and Table S2 in the **Online Supplementary Document**). More patients were hospitalised in the COVID-19 group (15.9%) than non-COVID-19 group (1.5%) (**Table 1**). Follow-up time was 3.3 months for the COVID group and 3.7 months for the non-COVID group (Figure S6 in the **Online Supplementary Document**).

**Table 1:** Clinical, sociodemographic characteristics and frequency of residual symptoms for COVID-19 and non-COVID-19 cases

Characteristics	COVID-19 cases (n = 412), n (%)	Non-COVID-19 cases (n = 402), n (%)	*P*-value*
**Age in years, median (IQR)**	36 (28-48)	34 (25-43)	<0.001
**Age group in years**			<0.001
15-30	126 (30.6)	167 (41.5)	
31-40	123 (29.9)	106 (26.4)	
41-50	75 (18.2)	82 (20.4)	
>50	88 (21.4)	47 (11.7)	
**Female sex**	228 (55.3)	259 (64.4)	0.01
**Number of acute symptoms, median (IQR)**	11 (8-14)	10 (7-13)	0.01
**Any comorbidities**	148 (35.9)	131 (32.5)	0.3
**Number of comorbidities**			0.6
0	264 (64.1)	271 (67.4)	
1	101 (24.5)	90 (22.4)	
>1	47 (11.4)	41 (10.2)	
**BMI in kg/m^2^, median (IQR)**	27 (23.5-29.8)	26.1 (23.5-29.1)	0.05
**Obesity**	102 (24.8)	86 (21.4)	0.7
Normal	310 (75.2)	316 (78.6)	
Obese (BMI = 30-34)	21 (5.1)	17 (4.2)	
Overweight (BMI = 35-39)	79 (19.2)	67 (16.7)	
Extremely obese (BMI>40)	2 (0.5)	2 (0.5)	
**Type of hospitalization**			<0.001
Outpatient (mild)	346 (84.2)	395 (98.5)	
Hospitalization, non-ICU (moderate)	59 (14.4)	6 (1.5)	
Hospitalization, ICU (severe)	6 (1.5)	0 (0)	
**At least one residual symptom >1 month after disease onset**	122 (29.6)	24 (5.8)	<0.001
**Number of residual symptoms**			<0.001
0	292 (70.9)	378 (94.0)	
1	57 (13.8)	9 (2.2)	
>1	63 (15.3)	15 (3.7)	
**Length of follow-up in months, median (IQR)**	3.35 (2.1-5.2)	3.65 (2.1-4.9)	0.5

BMI – body-mass index, IQR – interquartile range

*Pearson χ^2^ test or Wilcoxon rank sum test.

After one-month post-disease onset, 122/412 (29.6%) individuals from the COVID group reported at least one residual symptom, meeting the CDC’s definition of long COVID [15]. Residual symptoms were reported only by 24 (6%) individuals (**Table 1**) from the non-COVID group. Among 122 long COVID cases, 57 (46.7%) presented with more than two residual symptoms.

**Figure 2:**
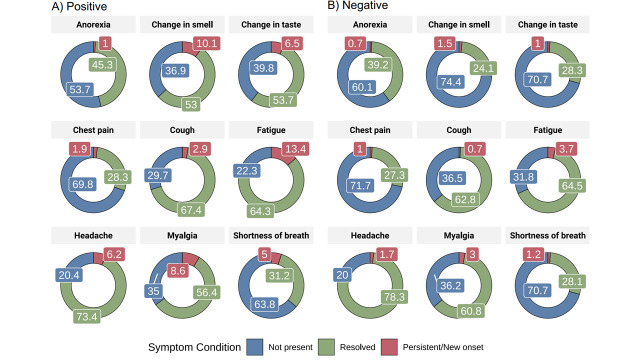
Frequency of acute and residual symptoms for COVID-19 and non-COVID-19 cases.

Within the COVID group, fatigue (n = 56, 13.7%), olfactory disorder (n = 42, 9.9%), myalgia (n = 36, 8.7%), gustatory disorder (n = 27, 6.6%), and headache (n = 26, 6.1%) were the most frequently reported residual symptoms (**Figure 2**), starting during the acute phase. Other symptoms not reported during the acute phase, such as memory loss and hair loss, were reported by patients with long COVID (Table S3 in the **Online Supplementary Document**). Compared to patients who recovered, the long COVID group had more women (63.1% vs 52.1%) and older individuals (40 (IQR = 32-51) vs 35 (IQR = 28-47) years; (**Table 2**)). Hospitalisation for disease was comparable between long COVID (n = 21/122, 17.2%) and recovered patients (n = 44/290, 15.2%).

**Table 2:** Clinical and sociodemographic characteristics for COVID cases with or without long COVID

Characteristics	No long COVID (n = 290), n (%)	Long COVID (n = 122), n (%)	*P*-value*
**Age in years, median (IQR)**	35 (28-47)	40 (32-51)	0.01
**Age group in years**			0.07
15-30	99 (34.1)	27 (22.1)	
31-40	85 (29.3)	38 (31.1)	
41-50	51 (17.9)	24 (19.7)	
>50	55 (19.0)	33 (27.0)	
**Female sex**	151 (52.1)	77 (63.1)	0.04
**BMI in kg/m^2^, median (IQR)**	26.9 (23.5-29.7)	27.2 (23.8-30.1)	0.8
**Number of acute symptoms, median (IQR)**	11 (7-14)	12 (9-15)	0.01
**Obesity**	70 (24.1)	32 (26.2)	0.4
Obese (BMI = 30-34)	12 (4.1)	9 (7.4)	
Overweight (BMI = 35-39)	57 (19.7)	22 (18.0)	
Extremely obese (BMI >40)	1 (0.3)	1 (0.3)	
**Number of comorbidities**			0.05
0	189 (65.0)	75 (61.0)	
1	75 (25.9)	26 (21.3)	
>1	26 (9.0)	21 (17.2)	
**Length of follow-up – months, median (IQR)**	3.30 (2.00-5.10)	3.75 (2.12-5.55)	0.07
**Required hospitalization or ICU**	44 (15.2)	21 (17.2)	0.27

BMI – body mass index, ICU – intensive care unit, IQR – interquartile range

*Pearson χ^2^ test or Wilcoxon rank sum test.

The risk of long COVID increased with age and was higher among individuals aged >50 years (odds ratio (OR) = 2.44; 95% CI = 1.29-4.66) compared to individuals aged 15-30. Individuals with more than five symptoms in the acute phase (OR = 3.15; 95% CI = 1.37-8.55) and females (OR = 1.55; 95% CI = 0.99-2.44) were more likely to develop long COVID (**Table 3**). After adjusting for the number of comorbidities, body mass index (BMI), respiratory allergy, and smoking in the sensitivity analysis, the results for the primary variables did not change significantly (Table S4 in the **Online Supplementary Document**).

**Table 3:** Multivariable analysis of odds ratio (lower and upper confidence intervals) for presenting long COVID symptoms

Characteristics	OR (95% CI)	*P*-value
**Age group in years, 15-30**	Reference	
31-40	1.71 (0.95-3.12)	0.078
41-50	2.04 (1.02-4.10)	0.045
>50	2.44 (1.29-4.66)	0.006
**Female sex**	1.55 (0.99-2.44)	0.055
**Number of symptoms (>5 acute symptoms)**	3.15 (1.37-8.55)	0.012

OR – odds ratio, CI – confidence interval

We also observed a co-occurrence of long COVID symptoms. At least 50% of individuals who reported fatigue also complained of gustatory dysfunction, anorexia, dysphonia, chest pain, headache, breathlessness, or myalgia, while 80% of participants with gustatory dysfunction also reported olfactory dysfunction (Figure S7 in the **Online Supplementary Document**).

Finally, we evaluated the impact of long COVID on the quality of life by administering the EQ-5D-3L questionnaire to 102 out of 122 patients with long COVID. Fifty-seven patients (55.8%) reported alteration in at least one dimension of QoL. The dimensions of pain and depression were the most impacted, with 42 patients (41.1%) reporting any degree of alteration (Table S5 in the **Online Supplementary Document**).

## DISCUSSION

We confirmed that the persistence of symptoms for more than one month following the acute phase was specifically associated with and frequently detected after SARS-CoV-2 infection. Risk factors for long COVID included female gender, being aged >50, and exhibiting more than five symptoms during the acute phase. Together, these findings demonstrate high frequency of long COVID, with well-defined demographic and clinical risk factors.

The proportion of patients with COVID-19 who developed long COVID varied from 7.5% to 89% [1-3,20]. Most studies only included patients who tested positive for SARS-CoV-2. One strength of our study is the inclusion of a comparator group of symptomatic patients, recruited at the same time/location, who tested negative by SARS-CoV-2 RT-PCR. Additionally, we evaluated most symptoms from the follow-up questionnaire at the time of recruitment in a blinded manner, prior to the SARS-CoV-2 test result. Including a control group is important, as many residual symptoms attributed to long COVID (such as fatigue and headache) are non-specific and could be triggered or aggravated by stress and psychosocial problems connected to the global health crisis [24-26]. We observed that non-confirmed COVID-19 cases reported similar residual symptoms after acute illness, but at a much lower frequency. Few studies have included a control group of symptomatic patients that tested negative for SARS-CoV-2 [4,27]. In a cohort of healthcare workers, olfactory disorders and hair loss were related to long COVID, while positive and negative cases both had similar frequencies of exhaustion/burnout and fatigue [27]. In a population-based digital retrospective cohort including outpatients and well-matched controls, 62 symptoms were associated with long COVID [4]. The inclusion of a control group is even more valuable for hospitalised cases, considering the increased risk of sequelae associated with iatrogenic procedures for treating severe disease [28]. Together, these findings highlight the importance of including a control group when determining the frequency of sequelae to avoid overestimating long COVID cases.

We observed a similar proportion of long COVID in groups with mild and moderate/severe disease [29,30]. We enrolled a small number of severe acute COVID-19 cases and could not conclude an association between disease severity and sequelae. Severe COVID-19 increases the risk of long COVID from 1.2 to eight times among hospitalised compared to non-hospitalised cases [31]. Nonetheless, the finding that long COVID occurs in a significant proportion of individuals after mild disease highlights its potential impact on the healthcare system, as COVID-19 variants and sub-variants continue to spread worldwide.

Fatigue was the most frequent sequelae and occurred isolated or associated with other clinical manifestations. As previously reported [32], we also observed a vast impact of long COVID on the quality of life. Although the mechanism is unknown, an exacerbation of the immunological response and multisystemic involvement [33] seems to be involved. Studies investigating the pathogenesis of long COVID are essential to improve treatment and attenuate the impact.

In line with previous studies [4,5,17,30,34-36], we found that long COVID was more prevalent in women and could be attributed to their greater propensity for going to a clinic [35]. Females represented 55% of the COVID group and 64% of the non-COVID group in our study. Sex hormone differences in immune response [37] and autoimmunity triggered by SARS-CoV-2 have been suggested to be involved in the disease pathogenesis of long COVID [36]. Other studies have found that individuals with more symptoms during the acute phase were at a higher risk for developing long COVID [17,27]. There are conflicting results regarding the role of age in the risk for developing long COVID. In a cohort of healthcare workers in Switzerland, most of whom had mild disease, young age was associated with a higher risk for developing long COVID [27], in contrast to our findings. Additional prospective studies including multicentre cohorts with larger sample sizes and older patients should be performed to evaluate if the risk for developing long COVID differs among age groups, racial groups, and ethnicities. Prospective studies to develop predictive models and algorithms should be performed for the early detection of long COVID cases.

Various organisations define long COVID differently. According to the CDC’s definition used in this study, it is the persistence of symptoms after four weeks post-onset [15]. Recently, a WHO-coordinated Delphi consensus set a period of more than three months after COVID-19 onset for the case definition of long COVID [38]. In our study, only 445 of the calls were performed three months after disease onset, which would mean that we detected long COVID in 68/216 (31.5%) of our patients. We also observed residual symptoms in 17/229 (7.4%) of the non-COVID-19 patients. As observed in our study, the prevalence of long COVID may vary between studies in the absence of a universal definition [6]. This impacts the estimation of the prevalence of long COVID cases globally.

This study has some limitations. Around 36% of patients were lost to follow-up, which may have influenced the estimates of residual symptoms. These patients had similar demographic and clinical characteristics to the included ones. Additionally, most participants were young with mild disease, so we cannot generalise our findings to the elderly population and patients with severe disease. Moreover, the participants were aware of their diagnosis of COVID-19 when questionnaire 3 was applied, which may have led to an overestimation of persistent symptoms. Finally, the short time to follow-up did not allow for an analysis of the duration of long COVID.

## CONCLUSIONS

We detected long COVID in 29.6% of patients with mild COVID-19 disease, with older age, female sex, and polysymptomatic acute disease as the main risk factors for persistent symptoms. Estimating the prevalence of long COVID is important for preparing healthcare systems to assist and guide these patients. Further studies should evaluate the effect of different variants of SARS-CoV-2 on long COVID and the potential impact of vaccines in reducing residual symptoms.

## Additional material


Online Supplementary Document
