# Supplementary material for: Prevalence and risk factors for long COVID after mild disease: A cohort study with a symptomatic control group
Source: J Glob Health. 2023 May 12;13:06015. doi: 10.7189/jogh.13.06015 (PMC10173895; doi:10.7189/jogh.13.06015)
Supplement: Online Supplementary Document [file jogh-13-06015-s001.pdf]

Supplementary Tables and Figures

Table S1 – Characteristics comparing loss of follow-up and included patients ….…… 2

Table S2 – Comorbidities for COVID-19 and non-COVID-19 cases ………………… 3

Table S3 – Other symptoms reported by patients with long COVID ……..…..……… 3

Table S4 – Sensitivity analysis of long COVID symptoms, adjusting for number of comorbidities, BMI, respiratory allergy and smoking ………………………………….. 4

Table S5 – Assessment of quality of life using EuroQol instrument EQ-5D-3L ……..... 5

Figure S1 – Prevalence of SARS-CoV-2 variants of concern in Bahia during the study period (from June, 2020, to April, 2021) ………………………………….………… 7

Figure S2 – Questionnaire 1 8

Figure S3 – Questionnaire 2 10

Figure S4 – Questionnaire 3 12

Figure S5 – Diagram of the questionnaire application: time and data collected …..… 13

Figure S6 – Time between residual symptoms call (questionnaire 3) and onset of symptoms ……………………………………………………………………………... 14

Figure S7 – Correlation matrix with the frequency of residual symptoms of long COVID patients ……………………………………………………………………………... 15

STROBE Statement – Checklist of items that should be included in reports of ***cohort studies*** ……………………………………………………………………………… 16

Table S1 – Characteristics comparing loss of follow-up and included patients.

Characteristic	Loss follow-up, N = 455	Study sample, N = 814	p-value*
**Age - years; median (IQR)**	37 (28-47)	35 (27-45)	0.10
**Female sex**	273 (60%)	487 (60%)	> 0.9
**Body Mass Index kg/m^2 (BMI); median (IQR)**	26.0 (23.5-28.9)	26.5 (23.5-29.4)	0.4
**Number of comorbidities**			> 0.9
0	296 (65%)	535 (66%)	
1	110 (24%)	191 (23%)	
> 1	49 (11%)	88 (11%)	
**SARS-CoV-2**			
Negative	217 (48%)	402 (49%)	0.6
Positive	238 (52%)	412 (51%)	
**Required hospitalization / ICU**			< 0.001
**Hospitalization non-ICU (moderate)**	12 (2.6%)	65 (8.0%)	
**Hospitalization-ICU (severe)**	5 (1.1%)	6 (0.7%)	

*Pearson's Chi-squared test; Wilcoxon rank sum test

Table S2 – Comorbidities for COVID-19 and non-COVID-19 cases.

Characteristic	Non-COVID-19 cases, N = 402[1]	COVID-19 cases, N = 412[1]	p-value*
**Hypertension**	45 (11%)	55 (13%)	0.3
**Diabetes Mellitus**	11 (2.7%)	12 (2.9%)	0.9
**Cardiac disease**	5 (1.2%)	10 (2.4%)	0.2
**Chronic pulmonary disease**	8 (2.0%)	5 (1.2%)	0.4
**Cancer**	2 (0.5%)	5 (1.2%)	0.5
**Immunodeficiency**	4 (1.0%)	2 (0.5%)	0.4
**Previous stroke**	1 (0.2%)	3 (0.7%)	0.6
**Other**	22 (5.5%)	17 (4.1%)	0.4
**Smoking**	43 (11%)	44 (11%)	> 0.9
**Missing**	4	5	

*Pearson's Chi-squared test; Wilcoxon rank sum test

Table S3 – Other symptoms reported by patients with long COVID.

Other reported residual symptoms	n (%)
Loss of memory	15 (12.3%)
Hair loss	9 (7.4%)
Joint pain	4 (3.3%)
Ear pain / ear disorder	4 (3.3%)

Table S4 – Sensitivity analysis of long COVID symptoms, adjusting for number of comorbidities, BMI, respiratory allergy and smoking.

Characteristic	Odds Ratio	95% CI	p-value
**Female sex**	1.56	0.99-2.49	0.059
**Number of acute symptoms**			
≤ 5	—	—	
> 5	3.46	1.48-9.54	0.008
**Age group – years**			
15 - 30	—	—	
31 - 40	1.66	0.91-3.06	0.10
41 - 50	2.07	1.02-4.24	0.044
> 50	2.24	1.13-4.48	0.021
**BMI kg/m^2; median**	0.99	0.93-1.05	0.7
**Number of comorbidities**			
0	—	—	
1	0.75	0.38-1.44	0.4
> 1	1.76	0.79-3.97	0.2
**Respiratory allergy**	1.32	0.78-2.21	0.3
**Smoking**	1.00	0.47-2.02	> 0.9

Table S5 – Assessment of quality of life using EuroQol instrument EQ-5D-3L.

Quality of Life[1]	Long COVID n = 102
**Mobility**	
I have no problems in walking about	94 (92.1%)
I have some problems in walking about	8 (7.8%)
I am confined to bed	0
**Self-care**	
I have no problems with self-care	96 (94.1%)
I have some problems washing or dressing myself	6 (5.9%)
I am unable to wash or dress myself	0
**Usual activities**	
I have no problems with performing my usual activities	73 (71.6%)
I have some problems with performing my usual activities	28 (27.5%)
I am unable to perform my usual activities	1 (0.9%)
**Pain / discomfort**	
I have no pain or discomfort	60 (58.8%)
I have moderate pain or discomfort	1 (0.9%)

I have extreme pain or discomfort	41 (40.2%)
**Anxiety / depression**	
I am not anxious or depressed	60 (58.8%)
I am moderately anxious or depressed	29 (28.4%)
I am extremely anxious or depressed	13 (12.7%)
**Score global (IQR)**	80 (70-90)

Figure S1 – Prevalence of SARS-CoV-2 variants of concern in Bahia during the study period (from June, 2020, to April, 2021).

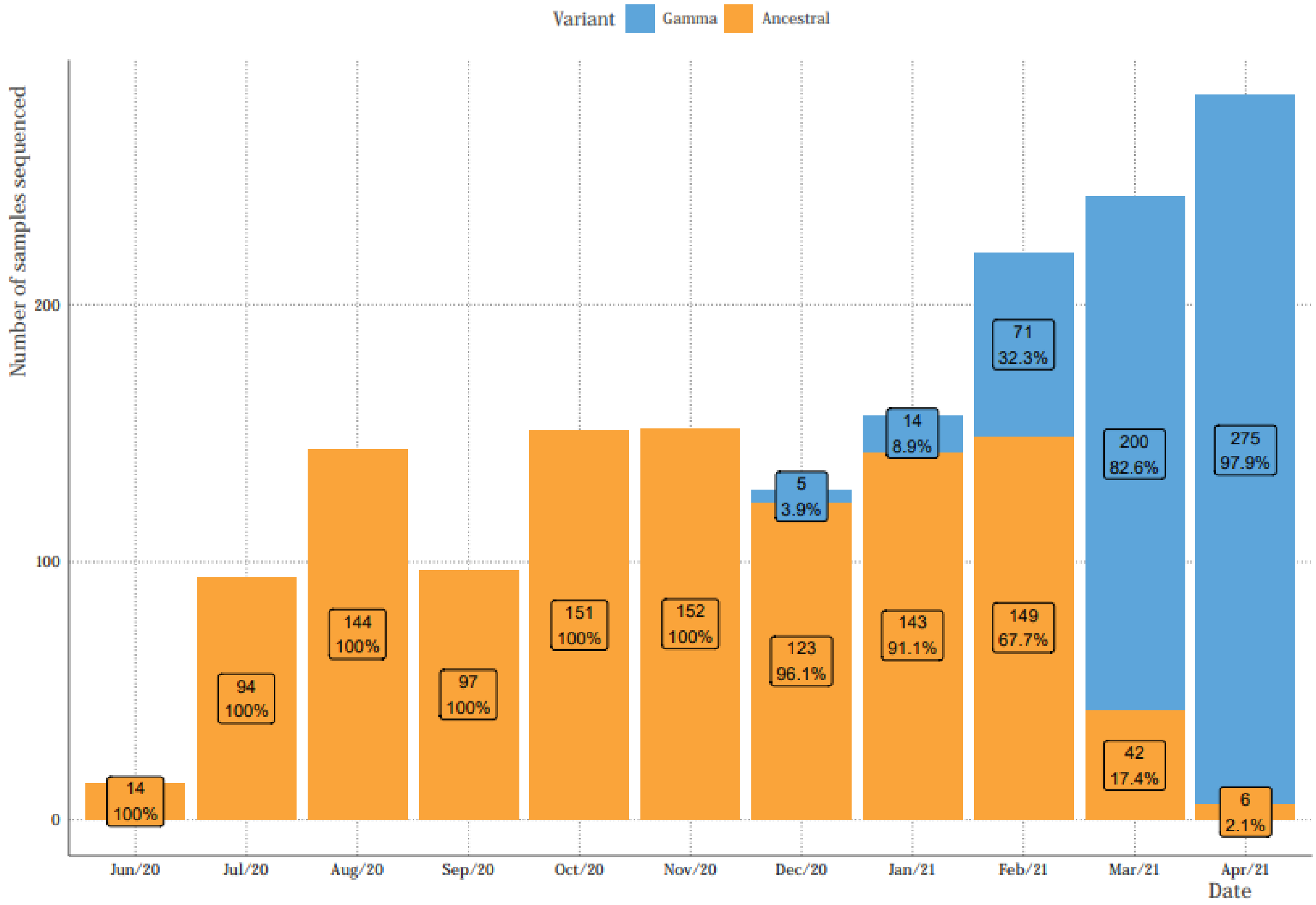

Data obtained from the Fiocruz Genomic Network [11].

Figure S2 – Questionnaire 1

Qual(is) foi o sintoma(s) inicial(is)?	☐ Falta de Ar<br>☐ Febre<br>☐ Tosse<br>☐ Alteração recente ao sentir cheiros<br>☐ Alteração recente ao sentir o gosto dos alimentos<br>☐ Coriza<br>☐ Espirro<br>☐ Fraqueza<br>☐ Dor no corpo<br>☐ Diarreira<br>☐ Enjoo<br>☐ Vômito<br>☐ Perda de Apetite<br>☐ Dor nas articulações<br>☐ Dor torácica<br>☐ Dor de cabeça<br>☐ Outros

Sintomas apresentados até o momento

	Não	Sim
Falta de ar	○	○
Nariz entupido	○	○
Coriza	○	○
Espirros	○	○
Dor na garganta	○	○
Dor na face	○	○
Dor de cabeça	○	○
Dor atrás dos olhos	○	○
Dor no ouvido	○	○
Dor muscular/no corpo	○	○
Dor nas articulações/Dor nas juntas	○	○
Dor no peito	○	○
Dor na barriga	○	○
Manchas no corpo	○	○
Fraqueza no corpo	○	○
Desconforto no peito	○	○
Perda de Apetite	○	○
Enjoo	○	○
Vômitos	○	○
Diarreia (2 ou mais episódios nas últimas 48h)	○	○
Sensação de pressão baixa ou desmaio	○	○
Alteração recente ao sentir cheiros	○	○

Alteração recente ao sentir o gosto dos alimentos	○	○
Febre	○	○
Tosse	○	○

Data de início alteração no olfato ______________________

Tempo em dias de alteração olfato ______________________

Data de início alteração no paladar ______________________

Tempo em dias alteração paladar ______________________

Está usando algum medicamento para febre? ○ Não ○ Sim

Qual medicamento para febre? ______________________

Está usando algum medicamento para tosse? ○ Não ○ Sim

Qual medicamento para tosse? ______________________

Usa algum medicamento diariamente/Tem alguma outra doença?? ○ Não ○ Sim

	Não sabe	Não	Sim
Corticoide Oral [Prednisona/Dexametasona/Prednisolona...]	○	○	○
Inibidor de bomba de Próton [Omeprazol/Pantoprazol/lansoprazol ...]	○	○	○
AINES [Aspirina/Ibuprofeno/Diclofenaco/Nimesulida/Meloxicam...]	○	○	○
Outros medicamentos	○	○	○

Qual outro medicamento ?
Listar separado por vírgula ______________________

IMC ______________________

Realizou exame para confirmação de infecção por COVID-19?	○ Não<br>○ Sim
Resultado PCR	○ Negativo<br>○ Positivo<br>○ Não realizado<br>○ Inconclusivo
Data do PCR	____________________
Tempo pós-sintomas do PCR	____________________
Resultado ELISA/Teste Rápido	○ Negativo<br>○ Positivo<br>○ Não realizado
Data do ELISA/Teste Rápido	____________________
Tempo pós-sintomas do ELISA	____________________

Figure S3 – questionnaire 2

Conseguiu realizar a Ligação de seguimento	○ Não<br>○ Sim
Data da tentativa	____________________
Motivo	○ Não atendeu<br>○ Número incorreto<br>○ Outro
Qual motivo	____________________
Data da ligação de retorno	____________________
Tempo pós sintomas da ligação de retorno	____________________
Necessidade de internação hospitalar?	○ Não ○ Sim
Internação	○ Ala/Enfermaria ○ UTI
UTI	○ Sem ventilação mecânica<br>○ Com ventilação mecânica
Situação atual	○ Saudável ○ Alguma sequela pós-covid<br>○ Óbito
Sintomas relatados na fase aguda	

**Sintomas durante o período da doença. Marcar os já relatados e qualquer novo**		
	Não	Sim
Febre	○	○
Tosse	○	○
Falta de ar	○	○
Nariz entupido	○	○
Coriza	○	○
Espirros	○	○
Dor na garganta	○	○
Dor na face	○	○
Dor de cabeça	○	○
Dor atrás dos olhos	○	○
Dor no ouvido	○	○
Dor muscular/no corpo	○	○
Dor nas articulações/Dor nas juntas	○	○

Realizado Ligação de acompanhameno de sequelas?	○ Não<br>○ Sim
Data da ligação de acompanhamento	____________________
Você sente/tem algum sintoma hoje?	○ Não<br>○ Sim
Tempo pós sintomas da ligação de sequela	____________________

**Sintomas presentes no momento da ligação**		
	Não	Sim
Febre Atual	○	○
Tosse Atual	○	○
Fadiga Atual	○	○
Falta de ar Atual	○	○
Dor de cabeça Atual	○	○
Dor no peito atual	○	○
Dor muscular/no corpo Atual	○	○
Falta de apetite Atual	○	○
Dificuldade de engolir (Atual)	○	○
Dificuldade para falar (Atual)	○	○
Alteração recente ao sentir cheiros (Atual)	○	○
Alteração recente ao sentir o gosto dos alimentos (Atual)	○	○

Outro sintoma persistente:	○ Não<br>○ Sim
Qual outro sintoma	____________________

Vômitos	○	○
Diarreia (2 ou mais episódios nas últimas 48h)	○	○
Sensação de pressão baixa ou desmaio	○	○
Alteração recente ao sentir cheiros	○	○
Alteração recente ao sentir o gosto dos alimentos	○	○

Figure S4 – questionnaire 3

Realizado Ligação de acompanhameno de sequelas?	○ Não<br>○ Sim
Data da ligação de acompanhamento	______________________
Você sente/tem algum sintoma hoje?	○ Não<br>○ Sim
Tempo pós sintomas da ligação de sequela	______________________

Sintomas presentes no momento da ligação

	Não	Sim
Febre Atual	○	○
Tosse Atual	○	○
Fadiga Atual	○	○
Falta de ar Atual	○	○
Dor de cabeça Atual	○	○
Dor no peito atual	○	○
Dor muscular/no corpo Atual	○	○
Falta de apetite Atual	○	○
Dificuldade de engolir (Atual)	○	○
Dificuldade para falar (Atual)	○	○
Alteração recente ao sentir cheiros (Atual)	○	○
Alteração recente ao sentir o gosto dos alimentos (Atual)	○	○

Outro sintoma persistente:	○ Não<br>○ Sim
Qual outro sintoma	______________________

Figure S5 – Diagram of the questionnaire application: time and data collected.

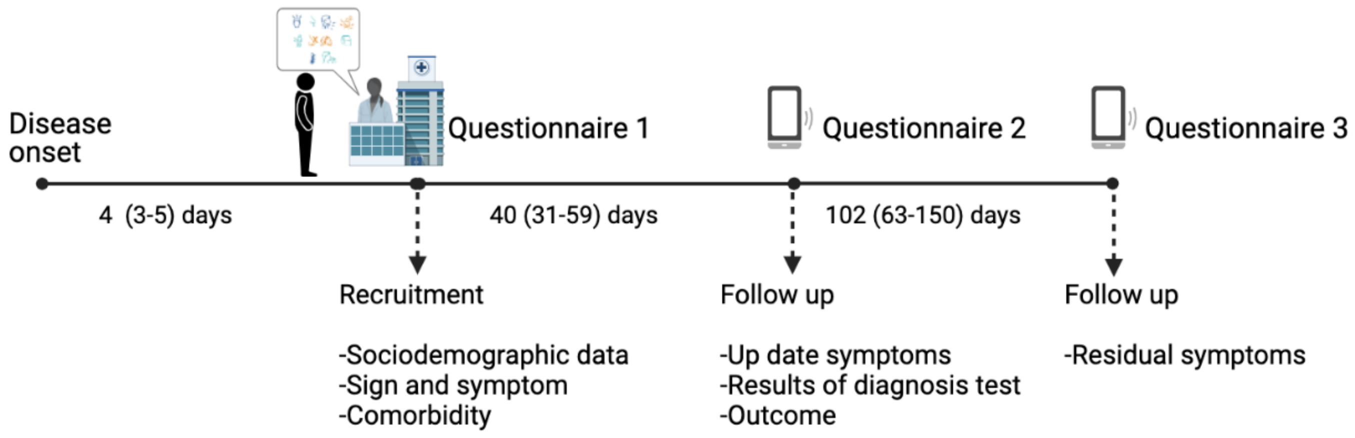

Figure created with BioRender. Available: https://biorender.com. Accessed: 28 April 2023.

Figure S6 – Time between residual symptoms call (questionnaire 3) and onset of symptoms.

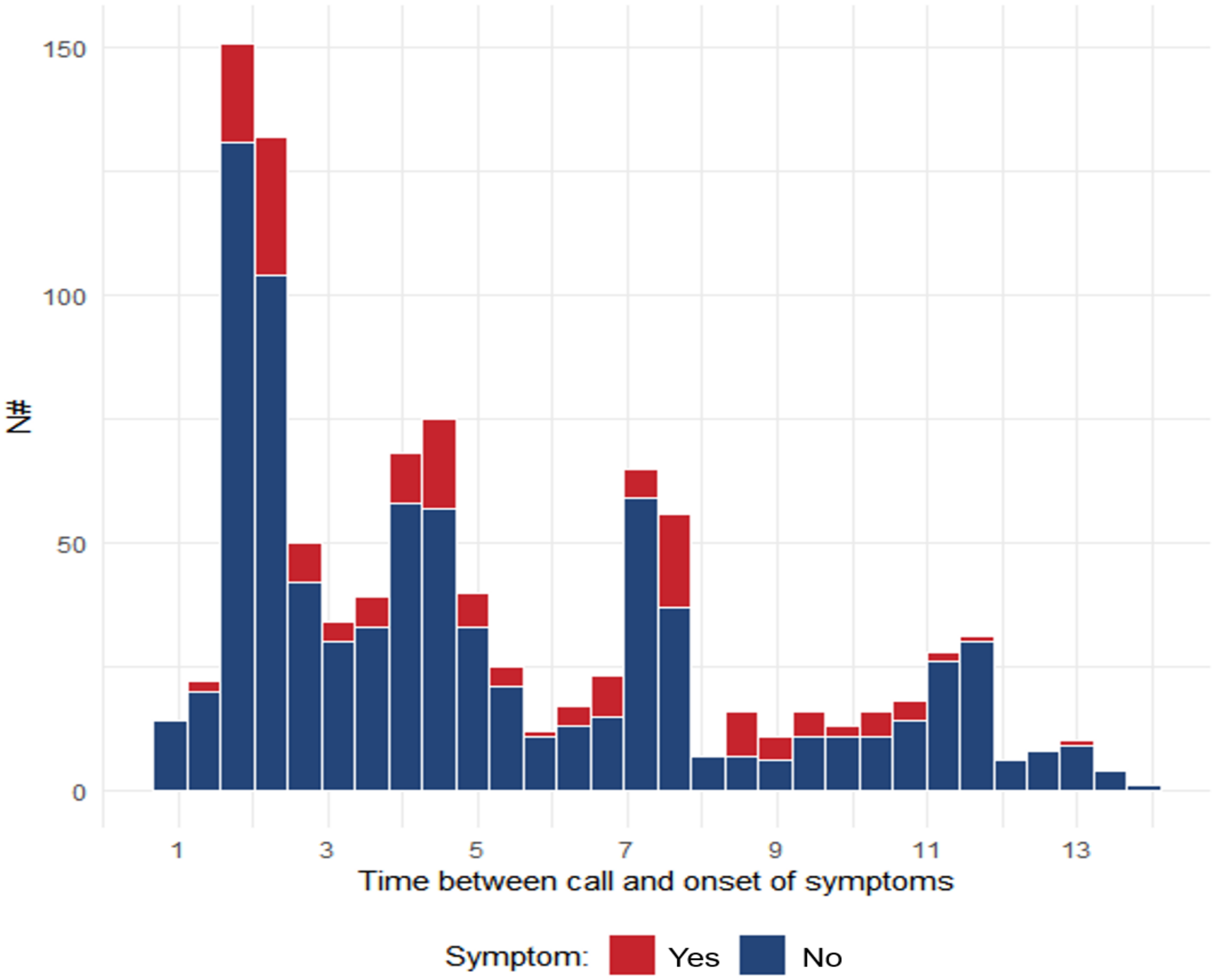

Figure S7 – Correlation matrix with the frequency of residual symptoms of long COVID patients.

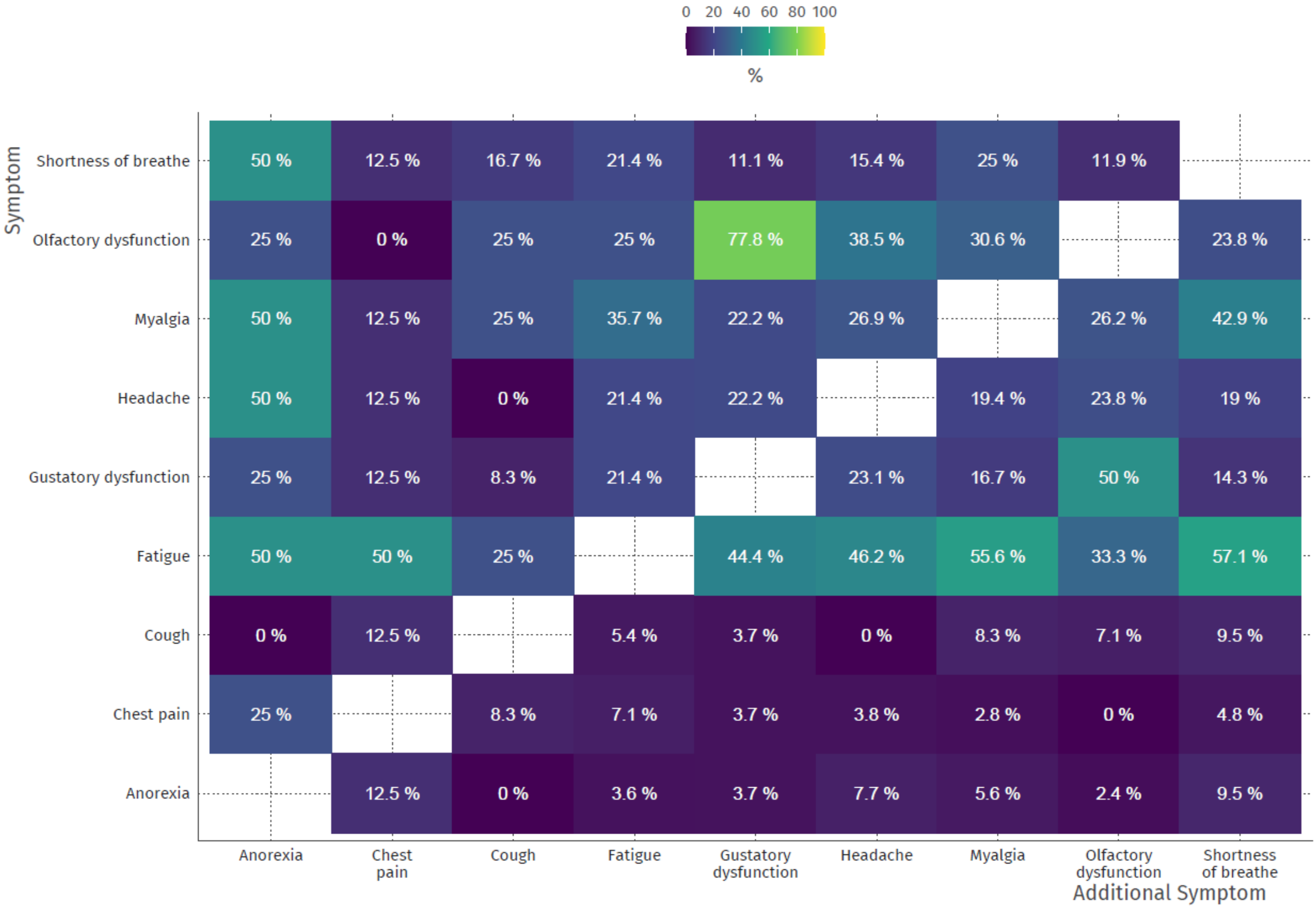

STROBE Statement — Checklist of items that should be included in reports of ***cohort studies***

	Item No	Recommendation
**Title and abstract**	1	(*a*) Indicate the study's design with a commonly used term in the title or the abstract – **section title**
		(*b*) Provide in the abstract an informative and balanced summary of what was done and what was found – **section abstract**
**Introduction**		
Background/rationale	2	Explain the scientific background and rationale for the investigation being reported – **section Background paragraph 1**
Objectives	3	State specific objectives, including any prespecified hypotheses **– section Background paragraph 2**
**Methods**		
Study design	4	Present key elements of study design early in the paper – **section Methods paragraphs 1-5**
Setting	5	Describe the setting, locations, and relevant dates, including periods of recruitment, exposure, follow-up, and data collection – **section Methods paragraphs 1-5**
Participants	6	(*a*) Give the eligibility criteria, and the sources and methods of selection of participants. Describe methods of follow-up – **section Methods paragraph 1**
		(*b*) For matched studies, give matching criteria and number of exposed and unexposed – **not appliable**
Variables	7	Clearly define all outcomes, exposures, predictors, potential confounders, and effect modifiers. Give diagnostic criteria, if applicable – **section Methods paragraphs 6-7**
Data sources/ measurement	8*	For each variable of interest, give sources of data and details of methods of assessment (measurement). Describe comparability of assessment methods if there is more than one group – **section Methods paragraphs 6-7**
Bias	9	Describe any efforts to address potential sources of bias – **section Methods paragraph 4**
Study size	10	Explain how the study size was arrived at – **not appliable**
Quantitative variables	11	Explain how quantitative variables were handled in the analyses. If applicable, describe which groupings were chosen and why – **section Methods paragraph 8**
Statistical methods	12	(*a*) Describe all statistical methods, including those used to control for confounding - **section Methods paragraph 8**
		(*b*) Describe any methods used to examine subgroups and interactions – **not appliable**
		(*c*) Explain how missing data were addressed – **not appliable**
		(*d*) If applicable, explain how loss to follow-up was addressed – **not appliable**
		(*e*) Describe any sensitivity analyses – **section Methods paragraph 8**
**Results**		
Participants	13*	(a) Report numbers of individuals at each stage of study—eg numbers potentially eligible, examined for eligibility, confirmed eligible, included in the study, completing follow-up, and analysed – **section Results paragraph 1**
		(b) Give reasons for non-participation at each stage – **section Results paragraph 1**
		(c) Consider use of a flow diagram – **Fig. 1**
Descriptive data	14*	(a) Give characteristics of study participants (eg demographic, clinical, social) and information on exposures and potential confounders – **section Results paragraph 1 / Table 1**

		(b) Indicate number of participants with missing data for each variable of interest – **section Results paragraph 1 / Additional file 1: Table S1**
		(c) Summarise follow-up time (eg, average and total amount) – **section Results paragraph 1 / Additional file 1: Fig.3**
Outcome data	15*	Report numbers of outcome events or summary measures over time – **section Results paragraphs 2,3**
Main results	16	(*a*) Give unadjusted estimates and, if applicable, confounder-adjusted estimates and their precision (eg, 95% confidence interval). Make clear which confounders were adjusted for and why they were included – **section Results paragraphs 4,5**
		(*b*) Report category boundaries when continuous variables were categorized
		(*c*) If relevant, consider translating estimates of relative risk into absolute risk for a meaningful time period – **not appliable**
Other analyses	17	Report other analyses done—eg analyses of subgroups and interactions, and sensitivity analyses – **not appliable**
**Discussion**		
Key results	18	Summarise key results with reference to study objectives – **section Discussion paragraph 1**
Limitations	19	Discuss limitations of the study, taking into account sources of potential bias or imprecision. Discuss both direction and magnitude of any potential bias – **section Discussion paragraph 7**
Interpretation	20	Give a cautious overall interpretation of results considering objectives, limitations, multiplicity of analyses, results from similar studies, and other relevant evidence – **section Discussion paragraphs 2-6**
Generalisability	21	Discuss the generalisability (external validity) of the study results – **section Discussion paragraph 6 and conclusions**
**Other information**		
Funding	22	Give the source of funding and the role of the funders for the present study and, if applicable, for the original study on which the present article is based – **section Declarations (funding)**

*Give information separately for exposed and unexposed groups.

Note: An Explanation and Elaboration article discusses each checklist item and gives methodological background and published examples of transparent reporting. The STROBE checklist is best used in conjunction with this article (freely available on the Web sites of PLoS Medicine at http://www.plosmedicine.org/, Annals of Internal Medicine at http://www.annals.org/, and Epidemiology at http://www.epidem.com/). Information on the STROBE Initiative is available at http://www.strobe-statement.org.
